# Molecular and virulence characterization of highly prevalent *Streptococcus agalactiae* circulated in bovine dairy herds

**DOI:** 10.1186/s13567-017-0461-2

**Published:** 2017-10-16

**Authors:** Maoda Pang, Lichang Sun, Tao He, Hongdu Bao, Lili Zhang, Yan Zhou, Hui Zhang, Ruicheng Wei, Yongjie Liu, Ran Wang

**Affiliations:** 1 Key Laboratory of Control Technology and Standard for Agro-product Safety and Quality, Key Lab of Food Quality and Safety of Jiangsu Province-State Key Laboratory Breeding Base, Institute of Food Safety and Nutrition, Jiangsu Academy of Agricultural Sciences, No. 50 Zhongling Street, Nanjing, 210014 China; 2 College of Veterinary Medicine, Nanjing Agricultural University, No. 1 Weigang, Nanjing, 210095 China

## Abstract

Bovine mastitis caused by *Streptococcus agalactiae* continues to be one of the major veterinary and economic issues in certain areas of the world. The more prevalent *S. agalactiae* strains that cause bovine mastitis in China dairy farms belong to a number of bovine-adapted sequence types (STs) ST67, ST103 and ST568. However, it is unknown why these STs can emerge as highly prevalent clones in bovine dairy farms. Here, to determine if a variety of virulence characteristics were associated with these highly prevalent STs, the molecular and virulence characterization of 116 strains isolated from bovine, human, fish and environment were analyzed. Our data showed that all bovine-adapted strains could be assigned to capsular genotype Ia or II, and carried pilus island 2b, and lactose operon. Importantly, we demonstrated that the growth ability in milk, biofilm formation ability and adhesion ability to bovine mammary epithelial cells (BMECs) were significantly higher for all bovine-adapted strains compared to strains from other origins. Additionally, ST103 and ST568 strains exhibited significantly higher hemolytic activity and cytotoxicity than ST67 strains. In conclusion, our study provides substantial evidence for the hypothesis that the virulence characteristics including efficient growth in milk, elevated biofilm formation ability, together with strong adhesion ability might have favored the high prevalence of the STs in the bovine environment, whereas the hemolytic activity and cytotoxicity were not the crucial characteristics.

**Electronic supplementary material:**

The online version of this article (doi:10.1186/s13567-017-0461-2) contains supplementary material, which is available to authorized users.

## Introduction

*Streptococcus agalactiae* is a gram-positive bacterium that has been considered as an important pathogen frequently associated with mastitis in bovine [1], neonatal meningitis in human [2] and meningoencephalitis in fish [3]. In large animals, bovine mastitis is the dominant health disorder leading to severe milk loss, and is responsible for significant financial losses in the dairy industry [4, 5]. An eradication has been implemented since 1960s to reduce the incidence of *S. agalactiae* mastitis in several European countries [6, 7]. However, in last several years, *S. agalactiae* mastitis reemerged and the occurrence frequency has increased again in Denmark, Norway and other Scandinavian countries [6, 7]. In other countries, especially in China, *S. agalactiae* has become one of the most frequently detected pathogens in cows diagnosed with subclinical mastitis [8]. Globally, bovine mastitis infected by *S. agalactiae* is still prevalent in many dairy farms worldwide [6].

Over the last decades, numerous studies have been conducted to investigate the molecular epidemiology [8–11] and genomic diversity of *S. agalactiae* [6, 12–14]. Studies using multilocus sequence typing (MLST) have shown that *S. agalactiae* belongs to corresponding sequence types (STs) with different host specificities [11, 15]. It has been reported that the prevalent strains circulated in China dairy farms are predominantly assigned to the bovine-adapted STs ST67, ST103 and ST568 [8]. ST67 belonging to clonal complex (CC) 67 has previously been considered to be the common ST among bovine isolates [16], and have been found in many countries including Brazil [17], France, the UK [18] and the USA [19]. ST103 was occasionally isolated from dairy cows in Brazil according to previous studies [17, 20], however, ST103 has emerged as a highly prevalent ST in bovine herds in Norway [7] and Denmark [11]. Both ST103 and ST568 belong to CC103, since ST568 is a single-locus variant (SLV, in which one allele differs from the ST) of ST103. To date, although ST568 was only detected in China, it was observed in high frequencies [8]. For human, isolates cluster five major CCs CC1, CC10, CC17, CC19 and CC23, which are separately from bovine isolates [12]. In fish farms, ST7 strains are found to be the major cause of streptococcosis outbreaks [13]. *S. agalactiae* was also observed in pond water [21], sediment [21] and feeding equipment [7], but no CCs were reported to be prevalent in specific environmental samples. Except for MLST analysis, comparative genomics has provided a comprehensive understanding of the distinct dynamic among *S. agalactiae* strains isolated from bovine, human and fish [6, 12–14]. The key role that horizontal gene transfer plays in the evolution of the bovine *S. agalactiae* strains has been highlighted [6, 12, 14]. It has been reported that human isolates are dominated to be few tetracycline resistant clones, and the acquisition of integrative and conjugative elements harbouring *tet*(M) may lead to the expansion of CC17 clones that then contributes to the increase of neonatal infections [12]. However, it is unknown why or how the bovine-adapted strains emerged as highly prevalent clones in dairy farms, and the mechanism of pathogenesis of these strains still remain limited.

Previous studies have shown that specific STs or strains often associate with the virulence characteristics which can contribute to their infection in host organisms [22–24]. Since the bovine-adapted STs (ST67, ST103 and ST568) are specifically responsible for the majority of *S. agalactiae* mastitis, it indicates that they may harbor some specific virulence characteristics which permit them to cause bovine mastitis and be prevalent within the bovine environment. Therefore, in this study, we sought to identify the virulence characteristics required for *S. agalactiae* infection in bovine mastitis, with the ultimate goal of elucidating the mechanisms underlying pathogenesis.

## Materials and methods

### Isolates collection and identification

A total of 116 *S. agalactiae* strains including 84 bovine strains and 32 reference strains isolated from human, fish and environment (soil, pond water and pond sediment) were collected in this study. The information (accession NO. in GenBank, biological resource, geographic resource and date of isolation) about the 116 strains is provided in Additional file 1. All bovine strains were recovered from milk samples taken from cows presenting clinical or subclinical mastitis between March 2011 and June 2016 from 14 bovine dairy farms in China. The 14 bovine dairy farms were located in eight cities, and were not epidemiologically related. Six bovine isolates belonging to the same STs were randomly taken from each dairy farms.

For isolation of bovine *S. agalactiae*, we diagnosed the bovine mastitis firstly. The clinical mastitis was determined through visual investigation by herd veterinarians, while the subclinical mastitis was evaluated using milk somatic cell counts (SCC) calculated by Fossomatic 5000TM automatic equipment (Foss Electric). Subclinical mastitis was suspected when SCC were greater than 1 000 000 cells/mL, but with no inflammation of the udder. Before the collection of milk samples, each teat was disinfected with swabs soaked in 70% ethyl alcohol, and the foremilk was discarded. Then, 20 mL milk from quarters with clinical or subclinical mastitis were collected, and 1 mL of milk sample was inoculated into 5 mL Todd-Hewitt broth (THB, BD Difco) and incubated at 37 °C for 6 h. After enrichment, the samples were streaked on Todd-Hewitt agar (THA, BD Difco) and incubated at 37 °C for 18 h. Subsequently, single colonies suspected to be *S. agalactiae* were isolated and the bacterial DNA was extracted using Bacterial DNA Kit (Omega). *S. agalactiae* were further identified by PCR amplification of 16S ribosomal DNA (rDNA) with universal primers 27F/1492R [25], and the sequence of 16S rDNA were deposited in GenBank (https://www.ncbi.nlm.nih.gov/genbank/).

### Genotypic characterization and phylogenetic analysis

Multilocus sequence typing was performed as previously described [26]. Briefly, seven housekeeping genes *adhP*, *pheS*, *atr*, *glnA*, *sdhA*, *glcK* and *tkt* from each strain were submitted to the *S. agalactiae* MLST database (http://pubmlst.org/sagalactiae/) to determine their identity against existing alleles. Each gene fragment was translated into a distinct allele, and each strain was classified into its ST by the combination of the alleles of the seven housekeeping genes. New STs that differed from the pre-existing STs were assigned new numbers and the data were deposited in the MLST database. The eBURST V3 program (http://eburst.mlst.net) was then used to identify the eBURST groups of 1148 STs deposited in *S. agalactiae* MLST database based on sharing of 6 out of 7 alleles using standard eBURST methodology [27]. The capsular genotypes Ia to IX of *S. agalactiae* were identified by a multiplex PCR assay developed by the previous study [28]. For phylogenetic analysis, the seven housekeeping genes previously sequenced were aligned and concatenated using MEGA7 (http://www.megasoftware.net/). Then, the phylogenetic tree was constructed using the neighbor-joining method on a set of 1000 bootstrap replicates [29].

### Distribution of virulence factors in *S. agalactiae*

A total of ten virulence genes *scpB*, *lmb*, *cylE*, *hylB, gapC*, *cspA*, *dltA*, *fbsA*, *fbsB* and *bibA*, and three pili genes designated as pilus island 1 (PI-1), PI-2a and PI-2b were detected according to previous study [21]. The examined virulence genes could be classified as being associated with bacterial adhesion and colonization (PI-1, PI-2a, PI-2b, *dltA*, *fbsA*, *fbsB*, *bibA* and *lmb*), bacterial invasion (*cspA*, *gapC* and *hylB*), immune evasion (*scpB*), toxin production (*cylE*), and metabolic adaptation (lactose operon) [5, 21]. The primers used to detect the lactose operon which referred to *lacABCDFEGX* [30] were newly designed in this study from conserved regions of *S. agalactiae* ATCC13813 and NEM316 using primer premier 6.24 (http://www.premierbiosoft.com/). All primers used in this study are listed in Additional file 2.

### Measurement of growth ability

The growth ability of each strain in milk was determined using the drop plate method [31]. In brief, cultures of each strain grown in THB for 6 h were adjusted to an optical density (OD) of 0.5 at 600 nm and then diluted 1:100 in 2 mL of sterile milk. The sterile milk inoculated with phosphate-buffered saline (PBS) was used as the negative control. Then, the inoculated milk was grown on a shaking incubator (80 rpm) at 37 °C for 12 h. For each time point, serial dilutions of milk were made in PBS and plated (100 μL in duplicate for each of the dilutions) on THA. After incubation for 24 h, colonies on THA were counted and the bacterial concentration was calculated. Experiments were carried out in triplicate and each independent experiment consisted of three technical replicates.

The growth ability of each strain in THB was determined as previously described [32] with some modifications. Briefly, cultures of strains grown in THB for 6 h were adjusted to an OD of 0.5 at 600 nm and then diluted 1:100 in 1 mL of THB. Every 200 μL of the inoculated cultures and blanks containing THB alone were injected into 96-well plates (Corning). Each growth condition was replicated in four wells and cultured at 37 °C. The growth of each strain was examined by measuring the absorbance of the cultures at 600 nm using a microplate reader (infinite M200 PRO, Tecan). The results are shown as the OD_600_ obtained at each time point, normalized against the background OD of THB alone. Each independent experiment was repeated in triplicate.

### Measurement of biofilm formation ability

The biofilm formation ability of *S. agalactiae* strains were evaluated by crystal violet staining using 96-well plates as described previously [33], with some modifications. Bacteria grown in THB for 6 h were normalized to an OD_600_ of 0.5 and then diluted 1:1000 in THB. The THB alone was used as the negative control. Two hundred microliters of inoculated THB was added to each well of 96-well plate and the plates were incubated statically at 37 °C. After incubated for 24 h, suspensions were discarded and the plates were washed with PBS thrice to remove planktonic cells. Subsequently, the surface-combined biofilms were fixed with methanol for 20 min. After discarding the methanol and drying at 37 °C, each well was stained with 200 µL of 1% (wt/vol) crystal violet solution for 15 min. Then, the plates were washed with PBS thrice to remove unbound dye. To quantify the biofilm biomass, the bound dye was redissolved in 200 µL of ethyl alcohol and a microplate reader was taken for each sample at OD_595_. Each strain was repeated in eight wells, and the assay was repeated in four independent occasions. The mature biofilm formed by *S. agalactiae* strains were also observed as described previously [34]. Briefly, 2 mL of the prepared 1:1000 dilution was added to each well of a 6-well plate (Corning) that contained a pre-sterilized microscopic glass slide as the substratum for biofilm growth. After incubation at 37 °C for 24 h for biofilm development, the planktonic bacterial cells on the glass slides were removed by washed using PBS. Then, 10 µL of Alexa Fluor 488 (Invitrogen) was added to each glass slide with a final concentration of 10 μg/mL and incubated in dark for 20 min. After washing triplicate using PBS, the biofilm formed on the slide was observed by Ultra View VOX (PerkinElmer).

### Measurement of hemolytic activity

The *S. agalactiae* strains were cultured on commercial Columbia blood agar plates (KeMaJia) for 24 h to determine their hemolytic phenotype firstly. Then, the hemolytic activity of all strains were measured using culture filtrates of bacteria cultured for 18 h as described previously [35]. In brief, every 200 μL of culture filtrates, which were adjusted to per mL of cell filtrate per 1 × 10^9^ CFU, were added to the well of a 96-well plate. Subsequentially, additions of 100 μL 2% sheep red blood cells (SRBCs) were added to the 96-well plate. The plate was incubated statically at 37 °C for 1 h, and then placed in 4 °C refrigerator for 12 h. Aliquots (100 μL) of normal saline and 1% (vol/vol) Triton-X 100 were added to an equal volume of 2% SRBCs as negative and positive controls, respectively. At the end of incubation at 4 °C, every 100 μL aliquot of supernatant was taken from each well to a new 96-well plate after centrifuged at 800 *g* for 10 min, and the OD of supernatant was measured at 540 nm. Hemolytic activity of each strain was reported as (*A* − *A*_*0*_/*A*_max_ − *A*_*0*_) × 100, whereas *A*_*0*_ is the absorbance caused by the background hemolysis occurring during incubation with normal saline, and *A*_*max*_ is the absorbance at 100% hemolysis after incubation with 1% Triton X-100 [36].

### Measurement of cytotoxicity

The release of lactate dehydrogenase (LDH) was used to determine the cytotoxic effect of *S. agalactiae* strains against bovine mammary epithelial cells (BMECs) using the CytoTox 96 Non-Radioactive Cytotoxicity Assay (Promega). In brief, BMECs cultured in 96-well plates were infected with 100 μL aliquots of bacterial suspensions at multiplicity of infection (MOI) of 1, 10 and 100 separately. BMECs lysed with the lysis solution were used as the positive control (100% cytotoxicity), whereas non-infected BMECs and bacteria alone were used as negative controls. The 96-well plates were centrifuged at 800 *g* for 10 min to move the bacteria to the surface of each monolayer before incubated for 3 h at 37 °C in 5% CO_2_. The plates were centrifuged again at 800 *g* for 10 min after incubation and 50 μL supernatant of each well was removed to new plates to determine the release of LDH by measuring OD_490_. Ultimately, percent cytotoxicity of each strain was calculated through the formula provided by the manufacturer’s instructions.

### Adhesion assay

Adhesion assay was performed according to the method described previously [37] with the following modifications. Cultures of each strain grown in THB for 6 h were harvested, washed twice with sterile PBS, and then resuspended in minimum essential medium (MEM, Yocon). BMECs cultured in 24-well plates (Corning) were infected with 500 μL aliquots of bacterial suspensions at an MOI of 100. The 24-well plates were centrifuged at 800 *g* for 10 min to move the bacteria to the surface of each monolayer before incubated for 2 h at 37 °C in 5% CO_2_. After incubation, the supernatants from each well were recovered and BMEC monolayers were washed thrice with PBS to obtain non-adherent bacterial suspensions. Subsequently, BMEC monolayers were trypsinized using 0.25% Trypsin–EDTA and further lysed with Triton X-100 at a final concentration of 0.1% (vol/vol) to obtain the adherent bacterial cells. The number of adherent bacterial cells and non-adherent bacterial cells from the supernatants were determined by plating serial dilutions on THA and counting the colonies after incubation for 24 h. The adhesion ability of *S. agalactiae* strains were expressed as percentage of adherent bacterial cells relative to the total number of adherent and non-adherent bacterial cells. Independent experiments were performed in triplicate.

### Statistical analysis

Continuous variables of virulence characteristics among different groups or subgroups were analyzed by analysis of variance (ANOVA), followed by Turkey’s and Dunnett’s T3 multiple comparison tests with *P* < 0.05 considered to be statistically significant, while *P* < 0.01 was considered to be an extremely significant difference. Error bars presented in the figures represent standard deviations of multiple replicate experiments. Data were collected and analyzed using Microsoft Excel (version 2010, Microsoft Corp.), GraphPad Prism (version 6.0, GraphPad Software Inc.) and SPSS Statics (version 22.0, SPSS Inc.).

## Results

### Genotypic characterization and phylogenetic analysis

**Figure 1:**
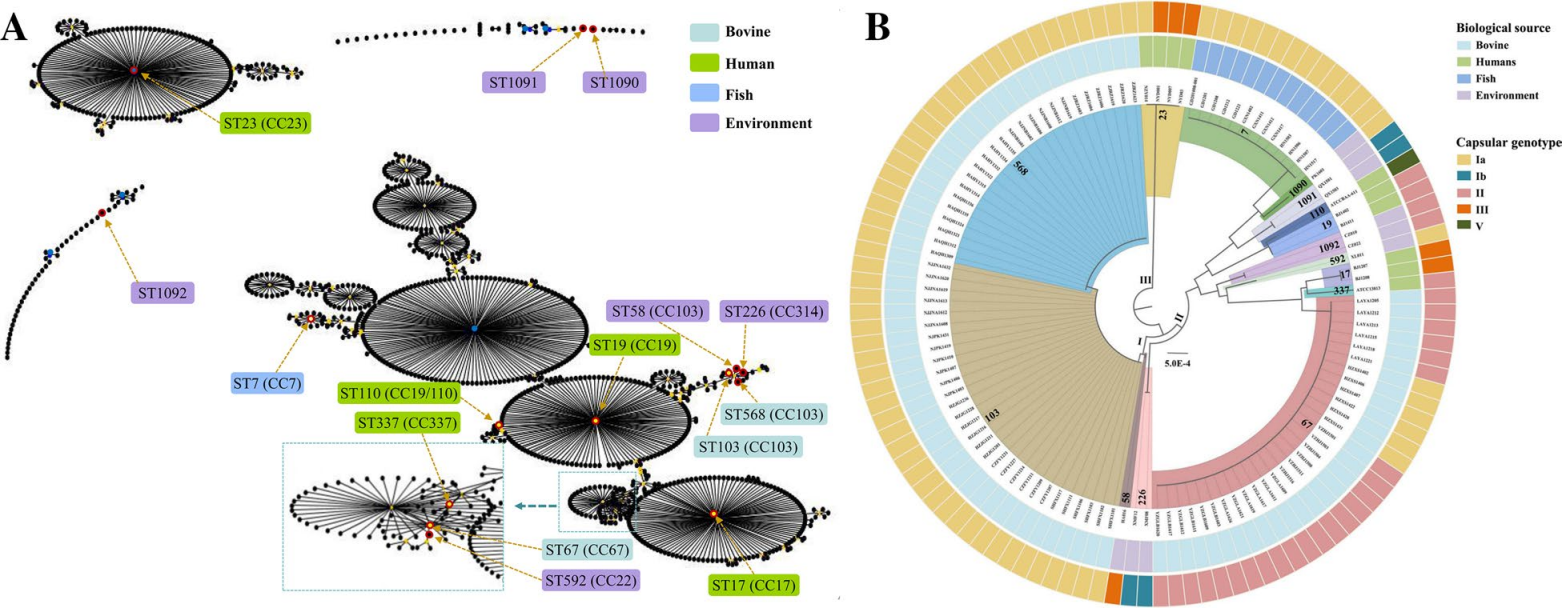
**Population snapshot generated by eBURST (A) and phylogenetic analysis of** ***S. agalactiae*** **strains (B)**.** A** A total of 1148 STs are displayed as single eBURST diagrams, and each ST is represented as a dot. The dots positioned centrally in the cluster are primary founders (blue) or subgroup founders (yellow). Red circles indicate the STs detected in this study and are marked by yellow arrows. **B** The internal color ranges refer to the different STs of the strains; the inner ring indicates the biological source, while the external ring indicates the capsular genotype.

The genotypic characteristics of the 116 *S. agalactiae* strains were analyzed using MLST and molecular capsular type (MCT). A total of 15 STs were identified, and the 84 bovine isolates were assigned to three STs, which were ST67 (n = 30), ST103 (n = 30) and ST568 (n = 24), respectively. The human isolates belonged to ST17, ST19, ST23, ST110 and ST337, while the fish isolates used in this study all belonged to ST7. Each of the environmental isolates was classified into a separate ST and three new STs (ST1090, ST1091 and ST1092) were identified based on new combinations of allele profiles. To illustrate the relationship between the 15 STs obtained from this study and the existing STs in *S. agalactiae* MLST database, an eBURST population snapshot was generated (Figure 1A, Additional file 3). The eBURST analysis showed these STs could be grouped as 10 groups and 57 singletons, and the large groups included a number of major subgroups that used to separate CCs. The bovine isolates were grouped in CC67 and CC103 (ST103 and its SLV ST568), and human isolates were grouped in CC17, CC19 (ST19 and its SLV ST110), CC23 and CC337. However, CC23 formed a distinct group, which was not related to CC17, CC19 or CC337. The fish isolates were grouped in CC7 (ST7). For environmental isolates, ST1090, ST1091 and ST1092 were identified as three singletons, while ST58, ST226 and ST592 were grouped in CC103, CC314 and CC22, respectively.

To further investigate the phylogenetic relationship of the 116 *S. agalactiae* strains, a phylogenetic tree based on the seven housekeeping genes was constructed. As shown in Figure 1B, the phylogenetic tree could be classified into three clades. Clade I was the largest cluster which including ST58, ST103 and ST568. Clade II was consisted of strains isolated from different origins, while Clade III was only consisted of four human isolates (ST23). To obtain further information on the genotypic heterogeneity of the individual STs, the capsular genotype was also analyzed. All strains belonging to ST7, ST103 and ST568 were assigned to capsular genotype Ia, whereas the ST67 strains were assigned to capsular genotype Ia or II. The capsular genotypes of human and environmental isolates were more diverse, which were consisted of Ia, Ib, II, III and V.

### Distribution of virulence factors in *S. agalactiae*

**Figure 2:**
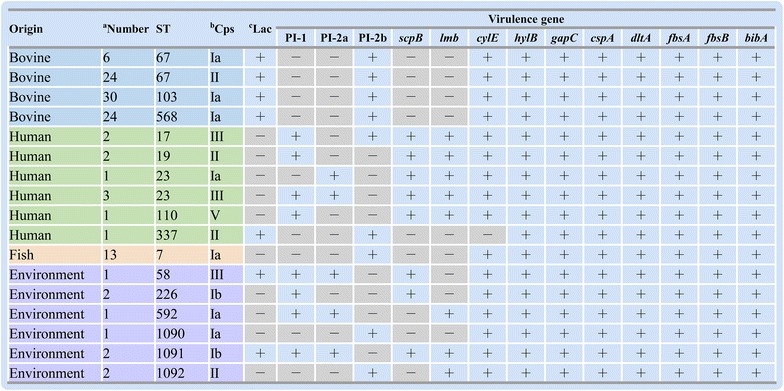
**Distribution of virulence factors in** ***S. agalactiae***. Only the representative genotypes were shown in the figure. ^a^Number represents the number of strains belonging to the same genotype; ^b^Cps and ^c^Lac represent the capsular genotype and lactose operon, respectively; The symbols “+” and “−” represent the presence or absence of virulence factors, respectively.

To determine whether *S. agalactiae* differed in virulence characteristics, an array of virulence genes were examined among the 116 strains. As shown in Figure 2, the examined virulence genes *hylB*, *gapC*, *cspA*, *dltA*, *fbsA*, *fbsB* and *bibA* were observed in all analyzed strains, while lactose operon (Lac), PI-1, PI-2a, PI-2b, *scpB*, *lmb* and *cylE* were responsible for the variety of virulence gene profiles. All the 84 bovine isolates belonged to virulence genotype Lac^+^PI-1^−^PI-2a^−^PI-2b^+^ *scpB* ^−^ *lmb* ^−^ *cylE* ^+^, and all fish isolates belonged to virulence genotype Lac^−^PI-1^−^PI-2a^−^PI-2b^+^ *scpB* ^−^ *lmb* ^−^ *cylE* ^+^. PI-2b was present in 100% of the bovine and fish isolates, whereas PI-1 and PI-2a were completely missing from those isolates. By contrast, PI-1 and PI-2a could be detected in human and environmental isolates. However, the virulence factors *scpB*, *lmb* and lactose operon distributed diversely in human and environmental strains. Most of the human isolates carried *scpB* and *lmb* genes, whereas only one isolate, ATCC13813, carried the lactose operon. In addition, *cylE* was found in all examined strains except for human isolate ATCC13813.

### Growth ability

**Figure 3:**
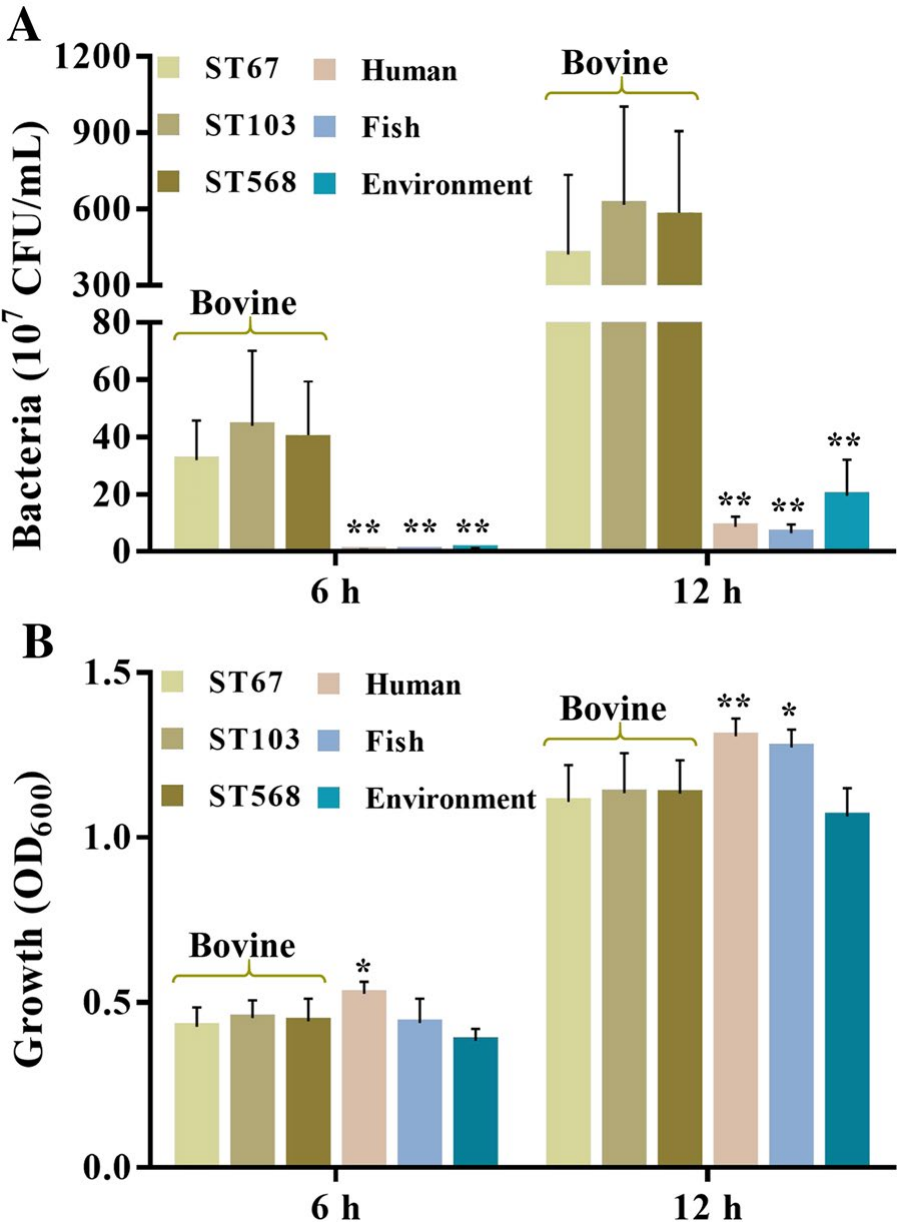
**Growth of** ***S. agalactiae*** **in milk (A) and THB (B)**.** A** The growth of *S. agalactiae* strains in milk were determined using the drop plate method; **B** the growth of *S. agalactiae* strains in THB were assessed by detecting the absorbance of bacterial cultures at OD_600_. * *P* < 0.05 or ** *P* < 0.01 indicates a significant difference compared to bovine group.

According to the origins, the 116 *S. agalactiae* strains were classified into four groups which were bovine, human, fish and environment, respectively. To determine whether there were significant differences among the bovine isolates belonging to different STs, they were further classified into three subgroups ST67, ST103 and ST568. The growth ability of all strains in milk were determined and being compared among different groups or subgroups. As depicted in Figure 3A, after cultured for 6 h, the bacterial concentration of bovine group was significantly higher than that of human, fish and environmental groups (*P* < 0.01). The concentration of each bovine subgroups cultured in milk was higher than 3.1 × 10^8^ CFU/mL, whereas none of the other groups was higher than 9.3 × 10^6^ CFU/mL. After cultured for 12 h, the bovine group increased further and showed an average bacterial concentration as high as 5.5 × 10^9^ CFU/mL. Similarly, bacterial concentrations of human, fish and environmental groups also increased, however, their concentrations were still significantly lower than that of bovine group (*P* < 0.01). Additionally, it was observed the bacterial concentration of subgroup ST67 was lower than subgroups ST103 and ST568, but without significant difference (*P* > 0.05). Unlike in milk, less variations were observed when *S. agalactiae* were cultured in THB. There was no significant difference in bacterial growth between bovine and environmental groups, when they were cultured in THB (Figure 3B). Moreover, the average bacterial growth of human and fish groups were even higher than that of bovine group after cultured for 12 h.

### Biofilm formation ability

**Figure 4:**
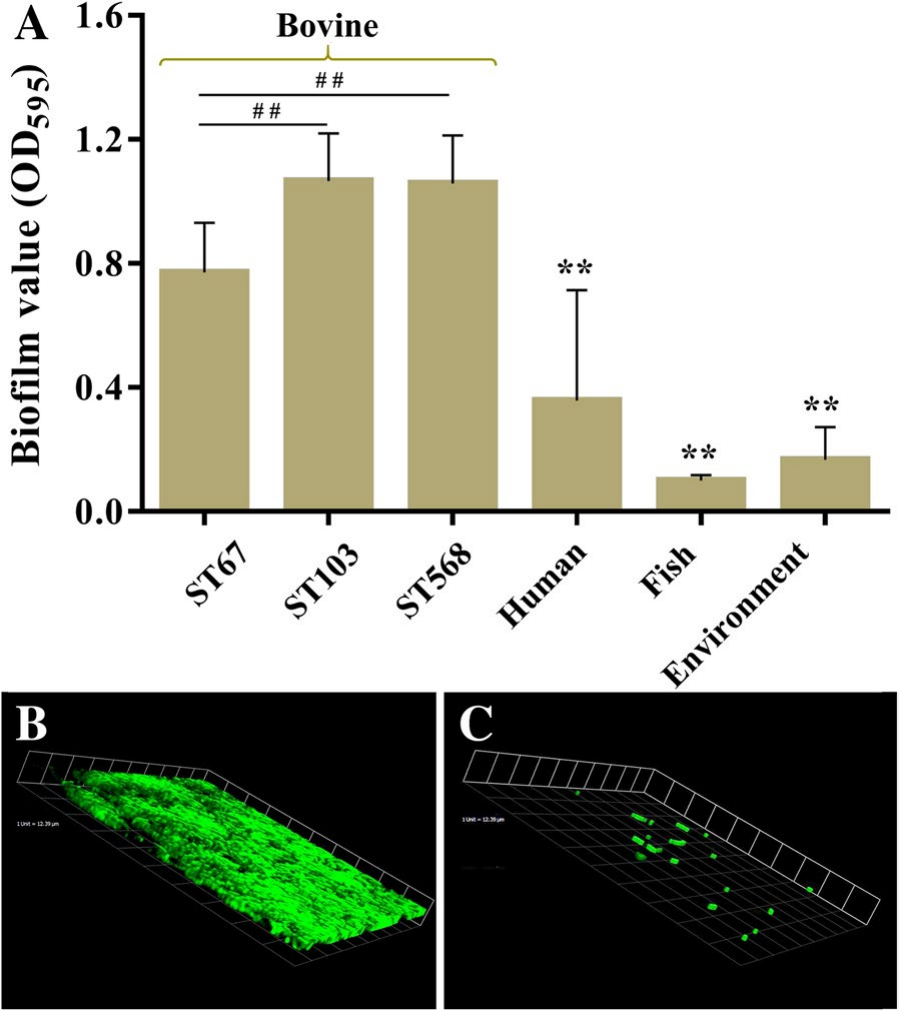
**Biofilm formation ability of** ***S. agalactiae*** **strains and CLSM images of biofilms**.** A** shows the biofilm formation ability evaluated by crystal violet staining using 96-well plates; **B** and **C** show the three-dimensional features of biofilms formed by strains NJPK1406 and GD201008-001, respectively. ^##^ *P* < 0.01 indicates a significant difference compared between subgroup ST67 and other two subgroups. ** *P* < 0.01 indicates a significant difference compared to bovine group.

The ability of each strain to form a biofilm was evaluated using crystal violet staining. As shown in Figure 4A, there were significant differences between bovine group and other three groups (*P* < 0.01). The subgroup ST103 formed the strongest biofilm, whereas fish group formed weakest biofilm. Furthermore, it was observed that although subgroup ST67 formed weaker biofilms compared to subgroups ST103 and ST568 (*P* < 0.01), subgroup ST67 could form much stronger biofilms than human, fish and environment groups (*P* < 0.01). However, it is noticeable that some human isolates, such as strains NZY014 and NYD001 belonging to ST23 could form a strong biofilm. The matured biofilms of representative strains NJPK1406 and GD201008-001, which belonged to ST103 and ST7 respectively, were observed using CLSM. As shown in Figure 4, the bovine isolate NJPK1406 formed structured multilayered aggregates of surface-adherent bacteria resembling a strong mature biofilm (Figure 4B), whereas the fish isolate GD201008-001 did not (Figure 4C).

### Hemolytic activity

**Figure 5:**
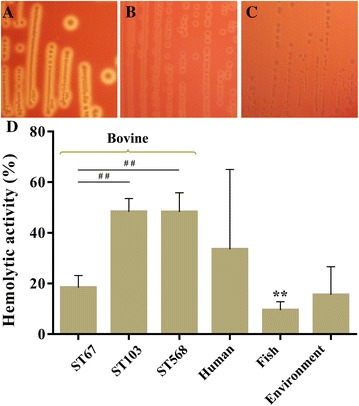
**Hemolytic phenotype and hemolytic activity of** ***S. agalactiae strains***. **A**, **B** and **C** show the hemolytic phenotypes of strains NJPK1406 (ST103), YZHJ1516 (ST67) and GD201008-001 (ST7), respectively; **D** shows the hemolytic activity of *S. agalactiae* strains. ^##^ *P* < 0.01 indicates a significantly different hemolytic activity between subgroup ST67 and other two subgroups. ** *P* < 0.01 indicates a significant difference compared to bovine group.

As shown in Figure 5A, different hemolytic phenotypes of *S. agalactiae* were observed after cultured on Columbia blood agar plates. Most of the strains belonging to subgroups ST103 and ST568 showed the complete hemolytic rings (Figure 5A), whereas ST67 strains predominantly showed weak hemolytic rings (Figure 5B). However, fish group and some strains of human and environmental groups showed incomplete hemolytic rings (Figure 5C). To further compare the hemolytic activity among different groups, the hemolytic activity was evaluated using a SRBCs lysis assay. Our result showed the hemolytic activity of each strain measured using culture filtrates was relatively low. As shown in Figure 5D, the hemoglobin released from SRBCs by most of the *S. agalactiae* strains were not greater than 50% of the value of the positive control. The subgroups ST103 and ST568 showed similar hemolytic activity with the hemolysis around 48.3%, whereas subgroup ST67 showed an average of 18.3% hemolysis. The fish group exhibited significantly lower hemolysis than bovine group (*P* < 0.01). Unlikely, no significant differences were found when human and environmental groups were compared to bovine group. In particular, the hemolytic activity of human strains were diverse with the lysis rate ranging from 2 to 68%.

### Cytotoxicity to BMECs

**Figure 6:**
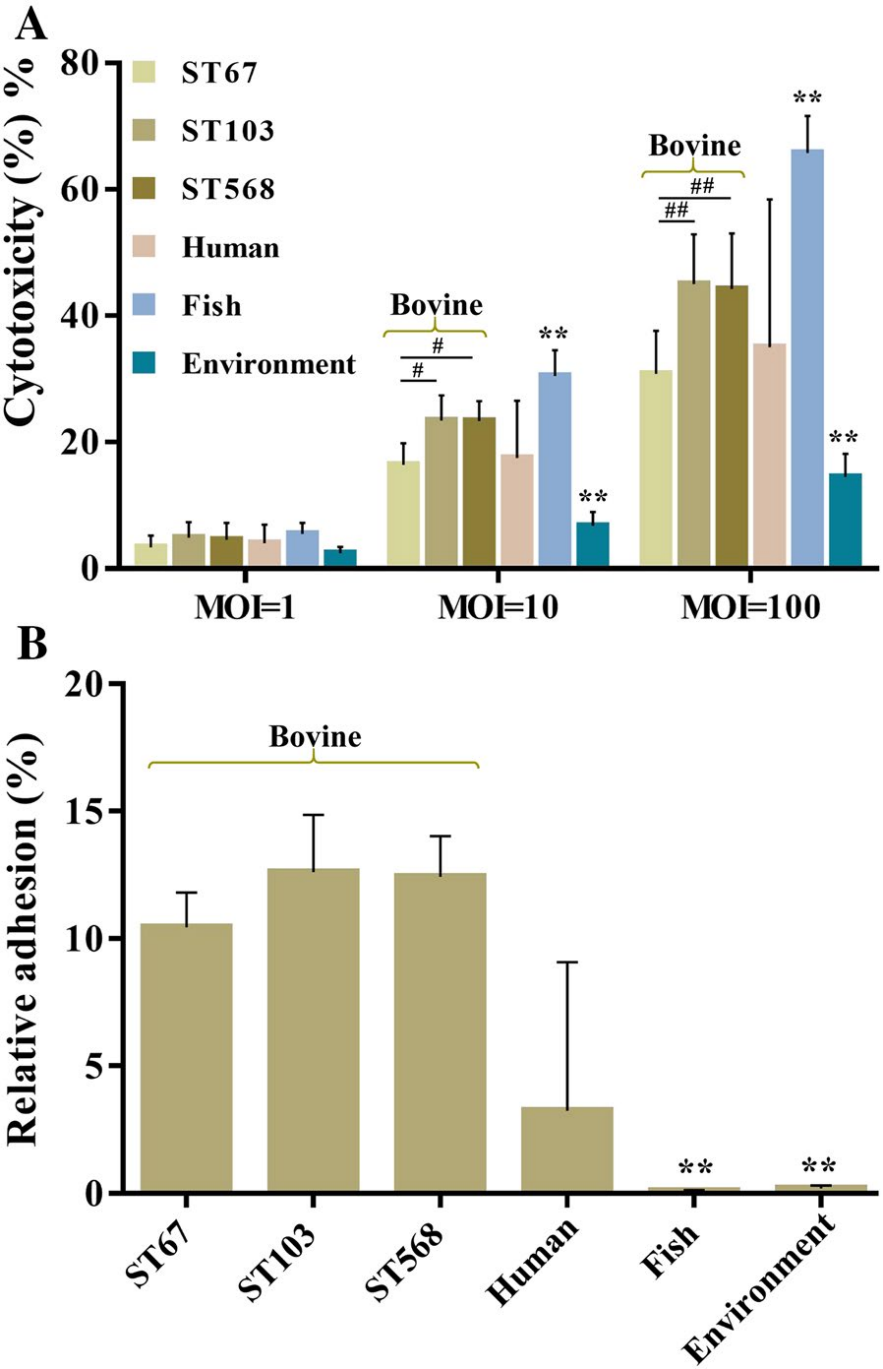
**Cytotoxicity and adhesion ability of** ***S. agalactiae*** **to BMECs**.** A** The percent cytotoxicity of each strain was determined by measuring LDH released from BMECs. **B** The relative adhesion of *S. agalactiae* strains were determined at an MOI of 100. ^#^ *P* < 0.05 or ^##^ *P* < 0.01 indicates a significant difference between subgroup ST67 and other two subgroups. ** *P* < 0.01 indicates a significant difference compared to bovine group.

To determine the cytotoxic effect of each *S. agalactiae* strain against BMECs, LDH release was examined, and the percent cytotoxicity was calculated. As depicted in Figure 6A, all groups were found to be cytotoxic to BMECs in a concentration-dependent manner, the cytotoxic effect become stronger along with the increase of MOI. No obvious cytotoxic effect of any group was found when MOI was 1. When at an MOI of 10, the percent cytotoxicity of fish group was significantly higher than bovine group (*P* < 0.01), whereas the percent cytotoxicity of environmental group was significantly lower than bovine group (*P* < 0.01). Significant differences were also found when subgroup ST67 was compared to subgroup ST103 or ST568 (*P* < 0.05), however, there was no significant difference between bovine and human groups on the cytotoxic effect. Furthermore, a similar result was also found when at an MOI of 100.

### Adhesion ability to BMECs

The adhesion ability of *S. agalactiae* strains to BMECs were evaluated. As shown in Figure 6B, all examined strains were able to adhere to BMECs after co-cultured for 2 h. However, there were significant differences between bovine group and other three groups. It showed that three bovine subgroups exhibited the strongest adhesion ability with the percent of adherent bacterial cells varying from 10.6 to 12.7%, whereas fish group showed the weakest adhesion ability. All of the bovine subgroups showed 100-fold higher levels of adherence than fish group (*P* < 0.01). Similarly, the adhesion ability of bovine group was also significantly higher than environmental group (*P* < 0.01). However, the adhesion ability of human strains were not uniformity, some strains such as NZY014 and NYD001 exhibited an adherence rate up to 12.1%, whereas adherence rates of other strains were lower than 3.2%.

## Discussion

Bovine mastitis caused by *S. agalactiae* continues to be one of the major veterinary and economic issues worldwide [6], representing a particularly prevalent problem in China [8]. In this study, we investigated the molecular characterization of 116 *S. agalactiae* strains isolated from bovine, human, fish and environment. Moreover, to identify the virulence characteristics of bovine-adapted strains which may confer them with ability to colonize and cause bovine mastitis, the virulence characteristics including growth ability in milk, biofilm formation ability, hemolytic activity, cytotoxic and adhesion to BMECs were analyzed.

To analyze the genotypic characteristics of the 116 strains, MLST, eBURST and phylogenetic analysis were used in this study. The results obtained from eBURST and phylogenetic analysis were similar. In general, the more alleles were identical, the STs would be more closer in the phylogenetic tree. The phylogenetic analysis showed bovine ST103 and ST568 formed a subclade, while bovine ST67 formed a subclade with human ST17 and ST337. It was also noted that human ST23 formed a clade which was clearly distinct to human ST17 and ST19. These results indicated that the STs which prevalent in specific animals may be genetically diverse. Notably, ST67 was the triple locus variants of ST17 and it has been proposed that ST17 and ST67 emerged from a common bovine ancestor [18]. However, our results showed bovine ST67 and human ST17 differed in the presence of lactose operon, PI-1, PI-2a, PI-2b, *scpB* and *lmb*. Thus, in agreement with Sorensen’s opinion [16], our data also suggested that human ST17 and bovine ST67 were distantly related. Since ST67, ST103 and ST568 were highly prevalent in bovine herds, it is intriguing to know which ST is more possible to cause clinical mastitis. However, since only few of the bovine strains were isolated from clinical cases (Additional file 1), the frequency analysis of STs linked with clinical or subclinical cases could not provide substantial data to determine which ST could cause a more severe infection, and this will be further investigated in our future work. Moreover, our results showed that MLST-based grouping did not correspond to grouping of strains based on MCT. The identical ST may be assigned to different capsular genotype, whereas the identical capsular genotype, such as genotype Ia, could be shared by various STs. This may be attributed to the lack of correlation between the two grouping methods since MLST is based on highly conserved housekeeping genes, whereas MCT is based on the horizontal transfer of capsular genes, which is likely to be supported by the acquired fitness or driven by the host immune response [9, 37].

To obtain more insight about the virulence factors of *S. agalactiae* strains, an array of virulence genes were screened (Figure 2). The result that *dltA*, *fbsA*, *fbsB, bibA, cspA*, *gapC* and *hylB* were found in all strains, suggesting they were conserved in *S. agalactiae* strains. The remaining seven virulence factors *lmb*, *scpB*, *cylE,* PI-1, PI-2a, PI-2b and lactose operon were responsible for the variety of virulence gene profiles among the examined strains. The absence of *lmb* and *scpB* in bovine and fish isolates suggests the activities of laminin-binding proteins and C5a peptidase may not be linked with their pathogenicity in bovine and fish. Similar to the findings of previous studies [8, 19], our data also showed that all bovine strains only carried PI-2b. Since previous works have reported that pilus-based vaccines can be used to prevent infections caused by *S. agalactiae* [38], the conserved PI-2b might be considered as a potential vaccine candidate for the development of subunit vaccines against bovine mastitis.

Successful survival and growth are requirements for bacteria to colonize the epithelial barrier, to produce virulence factors, and finally to transmit themselves to other hosts or the environment [39, 40]. To survive and colonize in the bovine mammary gland, it is of great importance for *S. agalactiae* to proliferate efficiently in milk. Importantly, we observed that all bovine isolates grew much faster than strains isolated from human, fish and environment (Figure 3A). After cultured for 6 h, the bacterial concentration of bovine group was higher than 100-folder compared to that of human and fish groups. However, the strains isolated from different origins showed less variation in THB (Figure 3B). After cultured for 12 h, the bacterial growth of human and fish groups were even higher than that of bovine group. These data suggest that growth ability in milk might contribute to the better adaptation for bovine isolates to the bovine herds. It has been reported that lactose operon can facilitate the metabolism of lactose [5], and the concentration of lactose contained in bovine milk can up to 5% [41]. Additionally, it was found that lactose operon was conserved in all bovine isolates. These data suggest that the ability of bovine isolates to grow rapidly in milk may attribute to the presence of lactose operon in bovine isolates. However, it should be noted that even some human and environmental isolates also carried lactose operon, they still grew much slower than bovine isolates in milk. These results indicate there might be some other genetic differences in bovine isolates except for lactose operon, which could contribute to the growth ability in milk.

Biofilm formed by bacteria are usually described as “a structured community formed by bacteria themselves enclosed in a self-produced polymeric matrix” [42]. In the mammary gland, the biofilm-like communities could facilitate microbial survival by enhancing resistance to antibiotics and clearance by host defense mechanisms [43, 44]. Our study showed the biofilm formation ability of bovine group was significantly stronger than that of other groups (*P* < 0.01). In addition, a positive correlation was found between biofilm formation and adherence to BMECs. The adhesion ability of bovine group to BMECs was also significantly stronger than other groups except for human group. For *Streptococcus* spp., the adhesion ability of bacterial cells to BMECs are important for the bacteria to colonize the lactating mammary gland despite the flow of milk, which results in excretion of planktonic bacterial cells [45]. Therefore, it is reasonable to speculate that the strong biofilm formation and adhesion abilities allow bovine-adapted isolates to colonize and persist in bovine mammary gland, where it is able to survive for long periods leading to chronic and subclinical mastitis. Previous studies have demonstrated that pili play important roles in biofilm formation and adhesion to epithelial cells [46–48], and the adherence could be inhibited by PI-2b protein-specific antiserum significantly [49]. Interestingly, our study shows that all bovine isolates carrying PI-2b alone exhibited strong biofilm formation and adhesion abilities, however, all fish isolates which also only carried PI-2b did not form intact biofilms and could hardly adhere to BMECs. This finding raises the question of whether PI-2b plays any role in the infection of fish *S. agalactiae*, which needs to further study.

The hemolytic activity and cytotoxicity of *S. agalactiae* strains were also examined in the present study. It was demonstrated that bovine group exhibited higher hemolytic activity than fish and environmental groups. However, there were striking differences between subgroup ST67 and subgroups ST103 and ST568 (*P* < 0.01), whereas there were no significant differences when subgroup ST67 was compared to other groups. Similar to hemolytic activity, subgroup ST67 also showed significantly lower cytotoxicity towards BMECs compared to both subgroups ST103 and ST568. These data suggest the hemolytic activity and cytotoxicity might not be the essential abilities for the infection of bovine-adapted *S. agalactiae*. However, the fish group exhibited significantly higher cytotoxicity than other groups, even though fish isolates showed the lowest hemolytic activity. These findings raised the intriguing question of the fish isolates could be highly cytotoxic to BMECs, but with extremely lower hemolytic activity. Conversely, it was reported that *S. agalactiae* strains with high hemolytic activity, were not cytotoxic to host cells [50]. Therefore, it is likely that differences underlying host–pathogen interaction at the cell–cell interface or unique virulence factors expressed in bacterial cells, may contribute to the cytotoxicity of fish isolates towards host cells.

Taken together, our study demonstrated that bovine isolates differed in the expression of some virulence-associated phenotypes compared to other isolates. In particular, bovine isolates all carried PI-2b and lactose operon, showed efficiently growth in milk, elevated biofilm formation ability and adhesion ability. Recently, studies have proposed that the evolutionary drove for bacteria to develop pathogenic characteristics was to access the nutrient resources that animals provided [39, 51]. For *S. agalactiae*, lethal outbreaks of ST7 strains have occurred in the fish farms in Asia, including China, Thailand and Kuwait [13], while ST17 strains has been well known as a hypervirulent clone occurred in pregnant women and newborns [52]. However, the bovine mastitis caused by *S. agalactiae* is usually chronic and subclinical, with intermittent episodes of clinical mastitis [53], and no available data in literature reported bovine isolates could be lethal for bovine. Generally, *S. agalactiae* is considered as a contagious pathogen which can readily be spread from the infected quarters to other quarters of the same cow, or from cow to cow [54]. Once the *S. agalactiae* strains could colonize and survive in bovine mammary gland, they could obtain nutrient-rich sources from milk to proliferate, and the harmful effect to bovine were long-term cumulative and might not be crucial. Therefore, we speculate that efficient growth in milk, elevated biofilm formation ability, together with strong adhesion ability might play key roles in the colonization and persistence in bovine mammary gland, whereas the hemolytic activity and cytotoxicity were not essential for the infection.

In conclusion, this study is a first step towards the elucidation of virulence strategies specific to the prevalent *S. agalactiae* STs. Although bovine ST67 were genetically diverse compared to ST103 and ST568, they harbored the similar virulence characteristics including growth ability in milk, biofilm formation ability and adhesion ability. The improved knowledge of the phenotypic characteristics of the prevalent strains is of crucial importance in the study of mastitis pathogenesis and would be valuable for the development of *S. agalactiae* control and treatment strategies. The ST-specific virulence characteristics permitting rapid colonization and persistence might explain why these STs could cause bovine mastitis and become prevalent within the bovine environment. However, the roles of these virulence characteristics in vivo and the host immune response merit further study.

## Additional files

**Additional file 1.** ***S. agalactiae*** **strains used in this study.**

**Additional file 2.** **Primers used in this study.**

**Additional file 3.** **eBURST analysis of 1148 STs deposited in** ***S. agalactiae*** **MLST database.**

## Abbreviations

*S. agalactiae*: *Streptococcus agalactiae*
ST: sequence type
BMECs: bovine mammary epithelial cells
MLST: multilocus sequence typing
CC: clonal complex
SLV: single-locus variant
SCC: somatic cell counts
rDNA: ribosomal DNA
THB: Todd-Hewitt broth
THA: Todd-Hewitt agar
PI: pilus island
Lac: lactose operon
OD: optical density
PBS: phosphate-buffered saline
SRBCs: sheep red blood cells
LDH: lactate dehydrogenase
MOI: multiplicity of infection
MEM: minimum essential medium
ANOVA: analysis of variance
MCT: molecular capsular type
