# Supplementary material for: Molecular and virulence characterization of highly prevalent Streptococcus agalactiae circulated in bovine dairy herds
Source: Vet Res. 2017 Oct 16;48:65. doi: 10.1186/s13567-017-0461-2 (PMC5644065; doi:10.1186/s13567-017-0461-2)
Supplement: Supplementary file 2 — Additional file 2. Primers used in this study. [file 13567_2017_461_MOESM2_ESM.doc]

**Additional file 2. Primers used in this study**

| **Target gene** | **Primer name** | **Sequence (5' to 3’)** | **Size of PCR**  **amplicon (bp)** | **Annealing**  **temp (℃)** | **Reference** |
| --- | --- | --- | --- | --- | --- |
| *adhP* | *adhp*-F | GGTGTGTGCCATACTGATTT | 672 | 50 | [1] |
|  | *adhp*-R | ACAGCAGTCACAACCACTCC |  |  |  |
| *pheS* | *phes*-F | ATATCAACTCAAGAAAAGCT | 723 | 49 | [1] |
|  | *phes*-R | TGATGGAATTGATGGCTATG |  |  |  |
| *atr* | *atr*-F | ATGGTTGAGCCAATTATTTC | 627 | 46 | [1] |
|  | *atr*-R | CCTTGCTCAACAATAATGCC |  |  |  |
| *glnA* | *glnA*-F | AATAAAGCAATGTTTGATGG | 589 | 50 | [1] |
|  | *glnA*-R | GCATTGTTCCCTTCATTATC |  |  |  |
| *sdhA* | *sdhA*-F | AACATAGCAGAGCTCATGAT | 646 | 48 | [1] |
|  | *sdhA*-R | GGGACTTCAACTAAACCTGC |  |  |  |
| *glcK* | *glcK*-F | GGTATCTTGACGCTTGAGGG | 607 | 48 | [1] |
|  | *glck*-R | ATCGCTGCTTTAATGGCAGA |  |  |  |
| *tkt* | *tkt*-F | ACACTTCATGGTGATGGTTG | 859 | 47 | [1] |
|  | *tkt*-R | TGACCTAGGTCATGAGCTTT |  |  |  |
| Capsular type | *cpsI-Ia-6-7*-F | GAATTGATAACTTTTGTGGATTGCGATGA |  | 54 | [2] |
|  | *cpsI-6*-R | CAATTCTGTCGGACTATCCTGATG |  |  |  |
|  | *cpsI-7*-R | TGTCGCTTCCACACTGAGTGTTGA |  |  |  |
|  | *cpsL*-F | CAATCCTAAGTATTTTCGGTTCATT |  |  |  |
|  | *cpsL*-R | TAGGAACATGTTCATTAACATAGC |  |  |  |
|  | *cpsG*-F | ACATGAACAGCAGTTCAACCGT |  |  |  |
|  | *cpsG*-R | ATGCTCTCCAAACTGTTCTTGT |  |  |  |
|  | *cpsG-2-3-6*-R | TCCATCTACATCTTCAATCCAAGC |  |  |  |
|  | *cpsN-5*-F | ATGCAACCAAGTGATTATCATGTA |  |  |  |
|  | *cpsN-5*-R | CTCTTCACTCTTTAGTGTAGGTAT |  |  |  |
|  | *cpsJ-8*-F | TATTTGGGAGGTAATCAAGAGACA |  |  |  |
|  | *cpsJ-8*-R | GTTTGGAGCATTCAAGATAACTCT |  |  |  |
|  | *cpsJ-2-4*-F | CATTTATTGATTCAGACGATTACATTGA |  |  |  |
|  | *cpsJ-2*-R | CCTCTTTCTCTAAAATATTCCAACC |  |  |  |
|  | *cpsJ-4*-R | CCTCAGGATATTTACGAATTCTGTA |  |  |  |
|  | *cpsI-7-9*-F | CTGTAATTGGAGGAATGTGGATCG |  |  |  |
|  | *cpsI-9*-R | AATCATCTTCATAATTTATCTCCCATT |  |  |  |
|  | *cpsJ-Ib*-F  *cpsJ-Ib*-R | GCAATTCTTAACAGAATATTCAGTTG  GCGTTTCTTTATCACATACTCTTG |  |  |  |
| PI-1 | *PI-1_F* | AACAATAGTGGCGGGGTCAACTG | 102 | 50 | [3] |
|  | *PI-1_R* | TTTCGCTGGGCGTTCTTGTGAC |  |  |  |
| PI-2a | *PI-2a_F* | CACGTGTCGCATCTTTTTGGTTGC | 128 | 50 | [3] |
|  | *PI-2a_R* | AACACTTGCTCCAGCAGGATTTGC |  |  |  |
| PI-2b | *PI-2b_F* | AGGAGATGGAGCCACTGATACGAC | 175 | 50 | [3] |
|  | *PI-2b_R* | ACGACGACGAGCAACAAGCAC |  |  |  |
| *cylE* | *cylE_F* | TTCTCCTCCTGGCAAAGCCAGC | 124 | 50 | [3] |
|  | *cylE_R* | CGCCTCCTCCGATGATGCTTG |  |  |  |
| *hylB* | *hylB_F* | TCTAGTCGATATGGGGCGCGT | 136 | 50 | [3] |
|  | *hylB_R* | ACCGTCAGCATAGAAGCCTTCAGC |  |  |  |
| *scpB* | *scpB_F* | TGAGCCTCAGGCATCGCACC | 109 | 50 | [3] |
|  | *scpB_R* | CCGCTGTCGATCAAGAGCACGG |  |  |  |
| *lmb* | *lmb_F* | TGGCGAGGAGAGGGCTCTTG | 105 | 50 | [3] |
|  | *lmb_R* | ATTCGTGACGCAACACACGGC |  |  |  |
| *cspA* | *cspA_F* | GGTCGCGATAGAGTTTCTTCCGC | 104 | 50 | [3] |
|  | *cspA_R* | AACGCCTGGGGCTGATTTGGC |  |  |  |
| *dltA* | *dltA_F* | GTTTTTGGTAGGGCAAACAGGGTGC | 100 | 50 | [3] |
|  | *dltA_R* | CGCAAATGTTGGCTCAACCGCC |  |  |  |
| *fbsA* | *fbsA_F* | AGTCACCTTGACTAGAGTGATTATT | 85 | 50 | [3] |
|  | *fbsA_R* | CCAAGTAGGTCAACTTATAGGGA |  |  |  |
| *fbsB* | *fbsB_F* | TCTGTCCAACAGCCGGCTCC | 144 | 50 | [3] |
|  | *fbsB_R* | TTCCGCAGTTGTTACACCGGC |  |  |  |
| *bibA* | *bibA_F* | AACCAGAAGCCAAGCCAGCAACC | 127 | 50 | [3] |
|  | *bibA_R* | AGTGGACTTGCGGCTTCACCC |  |  |  |
| *gapC* | *gapC_F* | AGACCGATAGCTTTTGCAGCACC | 100 | 50 | [3] |
|  | *gapC_R* | GATCCTTGACGGACCACACCG |  |  |  |
| Lactose operon | *lacI_F* | TAATGCTTTCGCAGTCGT | 756 | 50 | This study |
|  | *lacI_R* | GTGCTACTTGGGCAGGAT |  |  |  |
|  | *lacII_F* | ACAAATCGCACAAAGAGC | 702 | 52 | This study |
|  | *lacII_R* | CAACTACAGTATCAACACGAGAAT |  |  |  |
|  | *lacIII_F* | ATTGGTGGTGAGTGTCGT | 1374 | 50 | This study |
|  | *lacIII_R* | GGTGGCTTCTTGGTATTG |  |  |  |
|  | *lacIV_F* | AAGGCTAAGGCAGAAATA | 900 | 48 | This study |
|  | *lacIV_R* | TGGTAAAGGCTTGAATGT |  |  |  |

**References**

1. Jones N, Bohnsack JF, Takahashi S, Oliver KA, Chan MS, Kunst F, Glaser P, Rusniok C, Crook DW, Harding RM, Bisharat N, Spratt BG (2003) Multilocus sequence typing system for group B *streptococcus*. J Clin Microbiol 41:2530-2536

2. Imperi M, Pataracchia M, Alfarone G, Baldassarri L, Orefici G, Creti R (2010) A multiplex PCR assay for the direct identification of the capsular type (Ia to IX) of *Streptococcus agalactiae*. J Microbiol Methods 80:212-214

3. Kayansamruaj P, Pirarat N, Katagiri T, Hirono I, Rodkhum C (2014) Molecular characterization and virulence gene profiling of pathogenic *Streptococcus agalactiae* populations from tilapia ( Oreochromis sp.) farms in Thailand. J Vet Diagn Invest 26:488-495
